# Social Suppressive Behavior Is Organized by the Spatiotemporal Integration of Multiple Cortical Regions in the Japanese Macaque

**DOI:** 10.1371/journal.pone.0150934

**Published:** 2016-03-10

**Authors:** Naoya Oosugi, Toru Yanagawa, Yasuo Nagasaka, Naotaka Fujii

**Affiliations:** 1 Laboratory for Adaptive Intelligence, BSI, RIKEN, Wako, Saitama, Japan; 2 Graduate School of Arts and Sciences, University of Tokyo, Meguro, Tokyo, Japan; Università di Parma, ITALY

## Abstract

Under social conflict, monkeys develop hierarchical positions through social interactions. Once the hierarchy is established, the dominant monkey dominates the space around itself and the submissive monkey tries not to violate this space. Previous studies have shown the contributions of the frontal and parietal cortices in social suppression, but the contributions of other cortical areas to suppressive functions remain elusive. We recorded neural activity in large cortical areas using electrocorticographic (ECoG) arrays while monkeys performed a social food-grab task in which a target monkey was paired with either a dominant or a submissive monkey. If the paired monkey was dominant, the target monkey avoided taking food in the shared conflict space, but not in other areas. By contrast, when the paired monkey was submissive, the target monkey took the food freely without hesitation. We applied decoding analysis to the ECoG data to see when and which cortical areas contribute to social behavioral suppression. Neural information discriminating the social condition was more evident when the conflict space was set in the area contralateral to the recording hemisphere. We found that the information increased as the social pressure increased during the task. Before food presentation, when the pressure was relatively low, the parietal and somatosensory–motor cortices showed sustained discrimination of the social condition. After food presentation, when the monkey faced greater pressure to make a decision as to whether it should take the food, the prefrontal and visual cortices started to develop buildup responses. The social representation was found in a sustained form in the parietal and somatosensory–motor regions, followed by additional buildup form in the visual and prefrontal cortices. The representation was less influenced by reward expectation. These findings suggest that social adaptation is achieved by a higher-order self-regulation process (incorporating motor preparation/execution processes) in accordance with the embodied social contexts.

## Introduction

Human beings are social animals that manipulate behavioral repertoires depending on the social context generated by integration of environmental conditions and an individual’s past social experiences. We acquire this socially adaptive intelligence throughout our development. Social adaptation is a subjective internal process that is unique for each individual. However, regardless of the uniqueness of the internal mechanism, the expression of our social behavior is similar; therefore, there must be a common adaptive mechanism within the brain.

In monkey society, social adaptive behavior is also essential, and can be observed as suppressive behavior. When two monkeys are paired and share the same space, one monkey suppresses its action repertoires within the space to avoid violating the other monkey’s space. This suppressive function for avoiding social conflict is important for maintaining a stable social society [1]. To achieve this social suppressive behavior, a monkey must consider various social factors such as its own intentions and those of others [2], past experiences with others, its hierarchical status [3,4], what others are paying attention to, and the relative distance between individuals [5]. The monkey must integrate all these social factors, which provide the social context. Failure to integrate these social factors will lead to inappropriate social behavior.

Autistic humans often fail to perceive social information, such as emotion and intention, from others’ faces, which leads to disordered social communication [6,7]. To ensure appropriate social behavior, we must control our impulsivity during social interactions. It has been suggested that the prefrontal cortex is involved in the inhibitory control of impulsivity [8–10]. At the network level, a study of impulsivity in juveniles has shown that the strength of the connection between the premotor cortex and the attention network including the prefrontal cortex correlated with the controllability of impulsive behavior [11]. This finding suggests that we should consider the neural activity in the global brain network when trying to understand the neural mechanisms responsible for social suppressive behavior.

**Fig 1:**
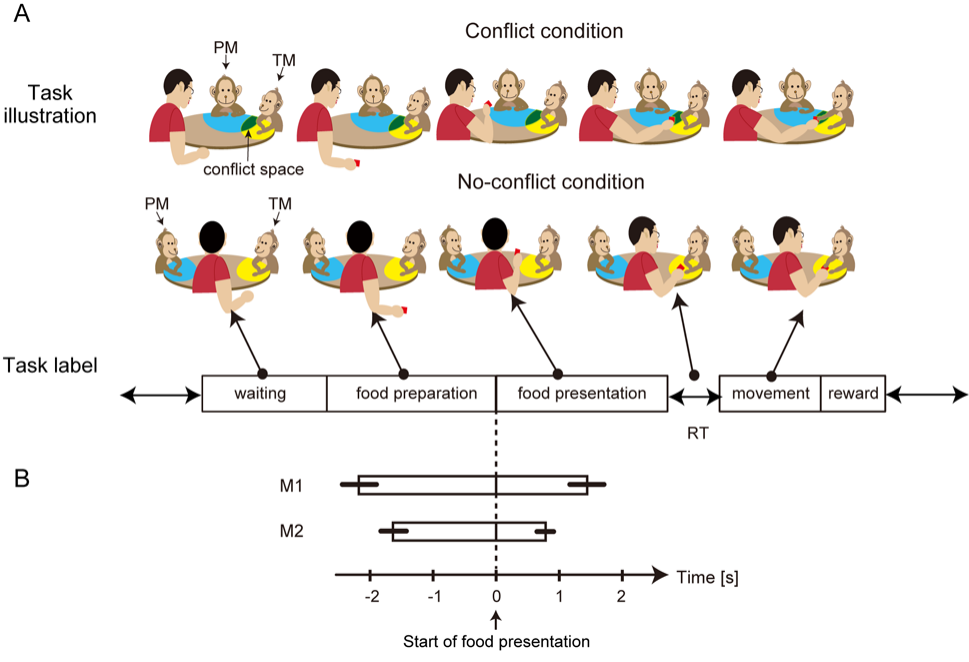
Schematic explanation of the social food-grab task. A. Task illustration: Schematic sequence of the food-grab task in the conflict and no-conflict conditions. The task had five periods: waiting, food preparation, food presentation, movement, and reward. TM, target monkey; PM, paired monkey. The spaces around the monkeys that were reachable only by the TM and PM are shown in yellow and blue, respectively. Green represents the conflict space, which was shared by the TM and PM. Task label: The labels of the five periods are shown under the Task illustration. RT, reaction time between the end of the food-presentation period and the onset of the movement period. B. Duration of the average food-preparation and food-presentation periods for M1 and M2 for their respective trials is shown as bars before and after the onset of the food-presentation period. The error bars indicate the SDs for the duration.

In previous studies of social suppressive behavior in the monkey, we recorded neural activity from the parietal [4] and prefrontal [3,4] cortices and the caudate nucleus [12] while monkeys performed a social food-grab task (see Fig 1A). In these studies, there were few restraints placed on the monkeys so that they could interact freely and show social adaptive behavior. Neurons from different areas of the brain exhibited different neural properties during the task. In the caudate nucleus, we found social context-dependent neural activity during the food-presentation period before the food-taking period in the social food-grab task [12]. Some neurons in the caudate nucleus exhibited increased neural activity during the food-presentation period, but the activity decreased when the monkey showed behavioral suppression. By contrast, the prefrontal baseline activity exhibited sustained context-dependent modulation [3]. These prefrontal neurons showed tonic baseline activity in the socially free condition, which did not require social adaptation, but showed a significant decrease or increase in baseline activity in the submissive or dominant mode, respectively. These findings suggest that adaptive social behavior is achieved by social context-dependent neural modulation.

These previous studies revealed that selected cortical and subcortical regions are involved in social context-dependent adaptive behavior. However, we do not know how the other areas are involved in this process, nor how social context is generated, represented, and used. To perform the food-grab task in a socially appropriate manner, monkeys must consider who the paired monkey (PM) is, its past experience with the PM, the relative location of the PM, and the PM’s intention at that moment. We hypothesized that this information is integrated and represented as social context in the brain, and is used as a reference for adaptive social behavior [13]. To investigate this idea further requires recording and analyzing the neural activity from large cortical areas simultaneously. By using electrocorticographic (ECoG) arrays, we recorded neural activity from most of the lateral cortical surface and a part of the medial prefrontal cortex while monkeys performed a social food-grab task. We found that the spatiotemporal patterns of neural dynamics correlated with social suppressive behavior.

## Materials and Methods

This study is reported according to the ARRIVE guidelines on animal research [14] (see S1 Text for details).

All experimental and surgical procedures were performed in accordance with the experimental protocols (No. H24-2-203(4)) approved by the RIKEN ethics committee and the recommendations of the Weatherall report “The use of non-human primates in research.” Implantation surgery was performed under sodium pentobarbital anesthesia, and all efforts were made to minimize suffering. Overall animal care was managed by the Research Resources Center at the RIKEN Brain Science Institute. Each animal was housed in a large individual enclosure with other animals visible and was maintained on a 12:12-h light:dark cycle with lights on at 8:00 am in a room with controlled temperature (25±2°C) and humidity (50±20%). The animals were given food (PS-A; Oriental Yeast Co., Tokyo, Japan) and water ad libitum, and daily fruit/dry treats as a means of enrichment and novelty. After every experiment, gummy candies and nuts were given as treats. The animals were occasionally provided with toys. The in-house veterinary doctor checked the animals and updated daily feedings to maintain weight. We attempted to offer as humane treatment of our subjects as possible. Five monkeys (*Macaca fuscata*) were included in the present study. Two were provided by the National BioResource Project of the Ministry of Education, Culture, Sports, Science and Technology (MEXT), Japan, and the other three were provided by Hamri Co. (Ibaragi, Japan). After completion of the study, one monkey with implanted ECoG electrodes was retired as an experimental animal. The other three monkeys, and the other monkey with implanted ECoG electrodes, participated in other studies; however, the ECoG electrodes have now been removed.

### Food-Grab Task

The food-grab task involved one human experimenter and two monkeys: a target monkey (TM), whose neural activity was recorded, and a PM. The human experimenter and the two monkeys sat around a round table with a diameter of 60 cm. In the task, the experimenter took a piece of food from a container placed under the table. The food was presented to the monkeys for a while and was then placed on the table (Fig 1A, Task illustration). In the conflict condition, there were three locations for food placement on the table (between the two monkeys (conflict space), between the experimenter and the TM, or between the experimenter and the PM (no-conflict spaces)). In the no-conflict condition, there were four locations of the food placement on the table (right and left sides of TM and PM, respectively) that did not overlap. The location of the food placement on the table was randomized in every trial so that the monkeys could not predict the location before the food was placed. During the task, there was no instruction regarding which monkey should take the food.

The food-grab task comprised five periods: “waiting,” “food preparation,” “food presentation,” “movement,” and “reward” (Fig 1A, Task label). When the monkey swallowed the food given in the previous trial, the next task was started. The waiting period was the period from the time when the task started to the time when the experimenter started to prepare the food. The food-preparation period was the period from the time when the experimenter started to prepare the food by grasping the food from the food container to the time when the experimenter’s hand appeared above the edge of the table. The food-presentation period was the period from when the experimenter’s hand appeared above the edge of the table to the time when the experimenter’s hand started to move to place the food on the table. During the food-presentation period, the experimenter held the food and kept showing the food for a while. The movement period was the period from the onset of the monkey’s hand movement to the time when the monkey started to eat the food. The reward period was the period from the time when the monkey started to eat the food to the time when the monkey swallowed the food. The onset and offset of each period were defined by analyzing the motion data from the experimenter’s hand and the monkeys’ arms. Each trial started when the experimenter confirmed that the monkey had finished chewing and swallowing the food given in the previous trial. The foods were boiled sweet potato, apple, or a small cookie. We randomly selected one of the three types of food for each trial to avoid the monkeys’ habituation.

### Task Condition

In the food-grab task, we manipulated two conditions: (1) the social hierarchy between the TM and the PM, and (2) the relative locations of the two monkeys. We prepared two TMs (M1 and M2). The age and weight of M1 and M2 were 9 years and 8.8 kg, and 6 years and 8.8 kg, respectively. To manipulate the TMs’ social hierarchical (submissive or dominant) status, each TM had two PMs. We prepared three monkeys who were not implanted with ECoG arrays as the PMs (M3–M5). The age and weight of M3–M5 were 8 years and 11.7 kg, 4 years and 7.1 kg, and 10 years and 10.8 kg, respectively. For M1, M3 was paired as the dominant monkey and M4 was paired as the submissive monkey. For M2, M5 was paired as the dominant monkey and M4 was paired as the submissive monkey. The hierarchical ranks were: M3 > M1 > M4 and M5 > M2 > M4. M1 and M2 were not paired in this study. We confirmed the hierarchy in preliminary experiments also involving the food-grab task. Data from the preliminary experiments were not included in the analysis in this study.

The other conditional manipulation was the relative spatial location of the TM and the PM. When the monkeys sat next to each other with a relative angle of 90° (conflict condition), they shared a space, which was called the “conflict space” (Fig 1A, Task illustration, green area). When the monkeys’ relative angle was 180° (no-conflict condition), there was no overlap in their peripersonal spaces, and thus no conflict (Fig 1A, Task illustration). In the conflict condition, there were two relative locations in which the PM sat to either the right or the left of the TM (right- and left-side conditions). We also set the location of the PM relative to the TM’s recording hemisphere, which we called the ipsilateral and contralateral conditions. In the no-conflict condition, there were two relative locations in which the experimenter sat to either the right or the left of the TM. In the analyses, we combined the right- and left-side trials for the no-conflict condition into one condition. In summary, the task comprised six conditions: two types of social hierarchy conditions × three types of spatial conditions.

### Duration of Task Periods

During the food-grab task, the experimenter subjectively controlled the duration of the food-preparation period and the food-presentation period. The averages and standard deviations of the durations are illustrated by the bar graph in Fig 1B. The average duration of the food-preparation periods for the recording sessions for M1 and M2 were 2136 ms (standard deviation (SD), 261 ms) and 1736 ms (SD, 222 ms), respectively. The average duration of the food-presentation periods for the recording sessions for M1 and M2 were 1352 ms (SD, 265 ms) and 730 ms (SD, 117 ms), respectively.

### Number of Trials

We performed the food-grab experiments over several successive days to obtain a sufficient number of trials for the analysis. The ECoG data had good stability over the experiment days [15]. M1 participated in 242 and 248 trials over 2 days, and M2 participated in 218, 219, and 215 trials over 3 days. Each day’s trials contained all six conditions: submissive or dominant, and ipsilateral conflict, contralateral conflict, or no conflict. The sequence of task conditions was randomized every day. For each submissive and dominant condition, the sequential order of ipsilateral conflict, contralateral conflict, and no-conflict conditions was randomized. All experiments were performed in the experimental laboratory during the daytime. Table 1 shows the number of trials for M1 and M2 under each combination of conditions.

**Table 1:** Number of trials in each social condition.

Subject	Social condition	Conflict–contralateral	Conflict–ipsilateral	No conflict
**M1**	Dominant	75	72	99
**M1**	Submissive	60	69	98
**M2**	Dominant	128	107	132
**M2**	Submissive	97	96	128

### ECoG Recording from the TM

**Fig 2:**
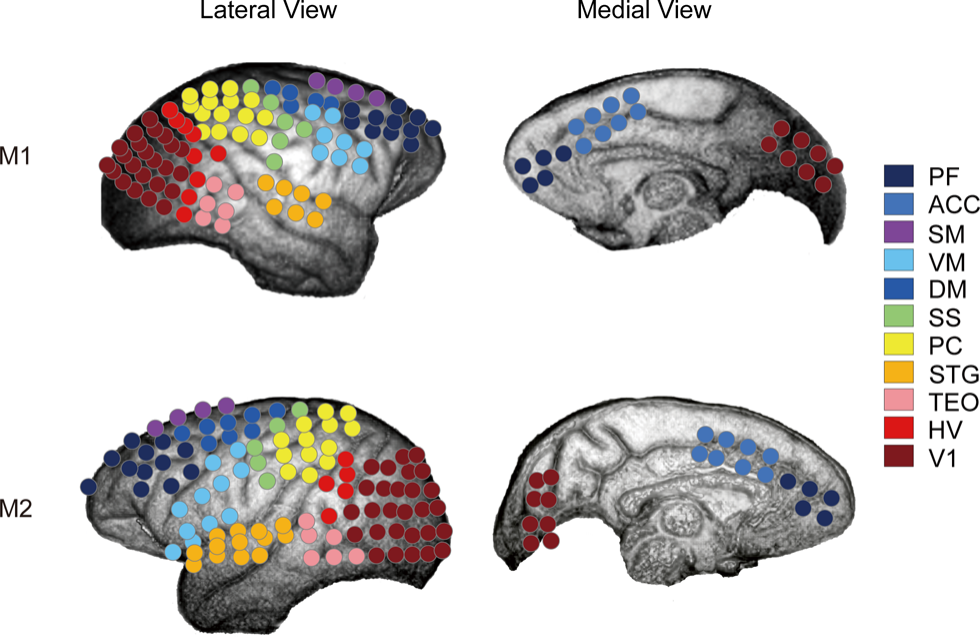
ECoG electrode arrays on the cortical surface of the TM. Electrode locations for M1 and M2 are indicated by circles on the lateral and medial views of a magnetic resonance image. The colors of the circles represent the cortical regions. PF, lateral and medial prefrontal cortices; ACC, anterior cingulate cortex; SM, supplementary motor cortex; VM, ventral primary and premotor cortex; DM, dorsal primary and premotor cortex; SS, somatosensory cortex; PC, parietal cortex; STG, superior temporal gyrus; TEO, posterior inferotemporal cortex; HV, higher visual cortex (V2 and V4); and V1, primary visual cortex.

We recorded ECoG signals from two monkeys (M1 and M2). Chronically implanted customized multichannel ECoG electrode arrays (Unique Medical Co., Tokyo, Japan) were used for the neural recordings [16]. The electrodes were made of 3-mm-diameter platinum discs that were dimpled at the center after being exposed to an insulating silicone sheet 0.8 mm in diameter. ECoG electrodes with 128 channels and an interelectrode distance of 5 mm were implanted into the subdural space of the left or right hemisphere of M1 or M2 (Fig 2). The details of the surgical implantation procedure are available on the Neurotycho.org public server (http://wiki.neurotycho.org/Surgical_Procedure). The monkeys were postoperatively treated with antibiotics (ampicillin, 5 mg/kg *bis in die* [BID]) for infection, cortisol (dexamethasone, 0.4 mg/kg BID) for brain edema, analgesics (butorphanol, 0.05 mg/kg BID) for surgical pain, and hemostatics (carbazochrome, 0.2 mg/kg BID) for bleeding. The physiological conditions of the monkeys were carefully monitored. They were given sufficient food, and their water consumption was monitored daily. If unable to consume an appropriate amount of water, the monkeys were intravenously or subcutaneously administered lactated Ringer’s solution.

Neural activity was recorded at a sampling rate of 1 kHz and was filtered with a 0.3–500 Hz band-pass filter using a Cerebus recording system (Blackrock Microsystems, Salt Lake City, UT, USA). In this study, neural activity was recorded from one monkey at a time, although the system for recording behavioral data could accommodate recording from multiple monkeys simultaneously. Electrode positions were identified postoperatively by combining magnetic resonance and X-ray images (Fig 2).

### Behavioral Data Recording

The behavior of the monkeys was monitored using a motion-capture system (Vicon, Oxford, UK) and a custom-made head-free eye-tracking system [16]. When using the motion-capture system, monkeys wore a custom-made elastic suit with reflective markers attached at the shoulder, elbow, and wrist. The three-dimensional locations of the markers were recorded and reconstructed in the subsequent offline analysis. The head-free eye-tracking system monitored corneal reflection of infrared rays, with the sampling rate set at 30 Hz. The right hand of the experimenter was also monitored using the motion-capture system. Other environmental information was stored using a conventional video camera. All neural, behavioral, and environmental information was captured synchronously and analyzed later.

### Other Settings

The monkeys sat on a primate chair during the performance of the task. The lower half of each monkey’s body was covered with an aluminum cover. Each had a collar fixed to a pole attached to the back of the primate chair. Other than these restraints, there was no behavioral restriction, so that the monkeys could move their eyes, head, arms, and torso freely during the task performance. The bottom of the primate chair was attached to a boom arm that could rotate around the table. The experimenter rotated the arm and locked the location of each monkey, so that the animals could not change their location by themselves.

### ECoG Data Analysis

Neural activity was recorded (band-pass filtered at 0.3–500 Hz) using 128-channel ECoG arrays placed on the cortical surface. Fig 2 indicates the locations of the electrodes in M1 and M2 overlaid on a magnetic resonance image. All signal processing was performed using the Signal Processing Toolbox and Statistics Toolbox in MATLAB (MathWorks, Natick, MA, USA).

ECoG data were sorted by the onset of the food-presentation period. The time series of ECoG data (–3,000 ms to 1000 ms around the onset of the food-presentation period) were extracted and converted to 250 ms time bins with 125 ms time shift (short “time bin”). A short-time Fourier transform with a Hanning window was applied to each short time bin to calculate the power spectrum for each condition, trial, and electrode. The frequency range was 4–160 Hz with 4 Hz resolution. Data obtained across two or three sessions (days) were combined and treated as single-session data without daily normalization because the signal quality was stable across the different days [15].

**Fig 3:**
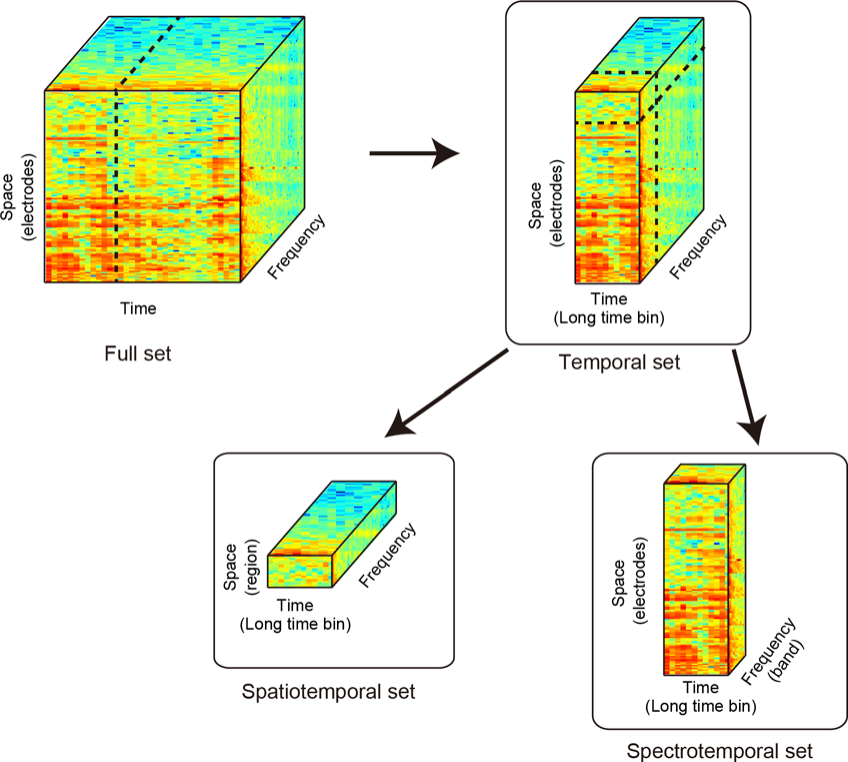
Schematic illustrations of the ECoG feature subsets. This figure shows how the tensor of the power spectrum of ECoG data was constructed. The ECoG feature tensor had three dimensions: space, time, and frequency. We named these three types of ECoG feature subsets (1) the temporal set, (2) the spatiotemporal set, and (3) the spectrotemporal set. Each ECoG feature subset was vectored for each trial and was used to construct social decoders to discriminate the social conditions. The accuracies of the social decoders were compared to identify which ECoG features contained information about the social conditions.

Using the short time bins, we defined a long time bin comprising several short time bins. The time series of ECoG activity was converted to long time bins of 1000 ms with a 125 ms time shift. The period from –3,000 ms to 1,000 ms had 25 long time bins, and each long time bin contained seven short time bins. Fig 3 shows the structure of preprocessed ECoG data (ECoG feature full set).

### Decoding Analysis of the Spatial Conditions

Traditional null hypothesis tests, such as the *t* test, may not be accurate when applied to multiple comparisons with large amounts of data [17]. One way to overcome this problem was to apply a modern multivariate analysis to the ECoG data. First, we applied a decoder analysis to determine how well the social conditions were represented in the ECoG power spectrum and quantified the information about hierarchical conditions by comparing the submissive and dominant conditions for each conflict–ipsilateral, conflict–contralateral, and no-conflict condition. We used a support vector machine (L2 norm, linear kernel, C value set to 1) as the neural decoder and evaluated its performance using a 50-fold cross-validation test [18]. The C value was fixed to compare the performance of decoders under the same condition. The decoders that predicted the social hierarchical conditions were generated in each spatial condition, and their performances were compared between spatial conditions to evaluate how well the social condition was represented. We did not compare the spatial conditions directly using the decoding analysis because it was difficult to distinguish the effects of the spatial factors from those of nonsocial factors such as the location of the food relative to the conflict space. The decoders were computed using the power spectrum of the ECoG data (Fig 3, full set), which included all electrodes, all frequencies, and 26 short time bins from –3000 ms to 375 ms around the onset of the food-presentation period (full-set neural classifier). We selected 375 ms as the upper limit of the time when the monkeys were waiting for the food delivery and had not yet started the reaching action.

### Decoding Analysis of the Social Conditions

We applied the decoding analysis to determine what kinds of neural features discriminate social conditions that guide social adaptation. We searched systematically to find the subset of ECoG features (see Fig 3) that contained the most information about social behavioral suppression. This method is similar to the searchlight method [19,20]. The aim of the searchlight method is to find a local feature space that contains rich information by continuously shifting the feature space. First, we grouped ECoG electrodes into 11 spatially discrete clusters based on the cortical sulcus (Fig 2). The electrode clusters did not overlap each other spatially. Fig 2 illustrates the spatial distribution of the ECoG electrode and cortical regions for monkeys M1 and M2.

Temporally, the ECoG power spectra were decomposed into long time bins of 1 s. The long time bin at each time point included seven short time bins of the ECoG power spectrum. The spectrum resolution was 4 Hz. The ECoG power spectrum data were analyzed separately for each of the three feature subsets: (1) a temporal set, (2) a spatiotemporal set, and (3) a spectrotemporal set (see Fig 3). Using these feature sets allowed us to construct three types of neural classifier: (1) a temporal neural classifier, (2) a spatiotemporal neural classifier, and (3) a spectrotemporal neural classifier. The temporal neural classifier was built for each time point using the power spectra of all regions and all frequencies. The temporal classifier was built to see how much information about social conditions was represented along the temporal sequence of the task. The spatiotemporal neural classifier was built for each time point using the power spectrum of the ECoG data of a single region and all frequencies. The spatiotemporal neural classifier was built to see how well each cortical region could discriminate social conditions along the temporal sequence of the task. The spectrotemporal neural classifier was built for each time point using the power spectrum of all electrodes and a single frequency. The aim in constructing the spectrotemporal neural classifier was to see how well each frequency domain could discriminate the social conditions along the temporal sequence of the task.

The chance level of the social condition classifier was determined using randomized label data. For example, in the conflict condition, the numbers of submissive and dominant conditions in M1 were 129 and 147, respectively. These condition labels were shuffled, and the accuracy of the social condition classifiers for the random label was calculated 50 times. The highest performance of the 50 results was set as the chance level.

### Decoding Analysis of the Motor Action

Decoding analysis was applied to determine whether neural features could discriminate motor actions. We pooled the trials in which the TM used its right and left hands to reach the food in the conflict conditions and constructed a neural classifier that discriminated whether the TM used its right or left hand. In this analysis, we built the temporal classifier only to see how much information about the motor plan was represented along the temporal sequence of the task.

### Decoding Analysis of the Reward Expectation

To extract neural representation of the reward expectation, we developed a classifier using an ECoG signal and recent outcomes as decoding features that predict whether the monkey could receive a reward. Decoding features were either a full set or a temporal set of ECoG spectrum data at a certain time window and the results (succeeded or failed) of the last three trials separated into binary categorical variables.

## Results

### Behavioral Data

The behavioral evidence for the hierarchical effects was investigated in three ways. Before the analysis, we excluded trials where the angle between the TM’s gaze direction and a line from the center of the TM’s body to the center of the table was greater than 60° because, in this case, the TM was not presumed to be engaged in the task. The number of excluded trials averaged three each day.

**Fig 4:**
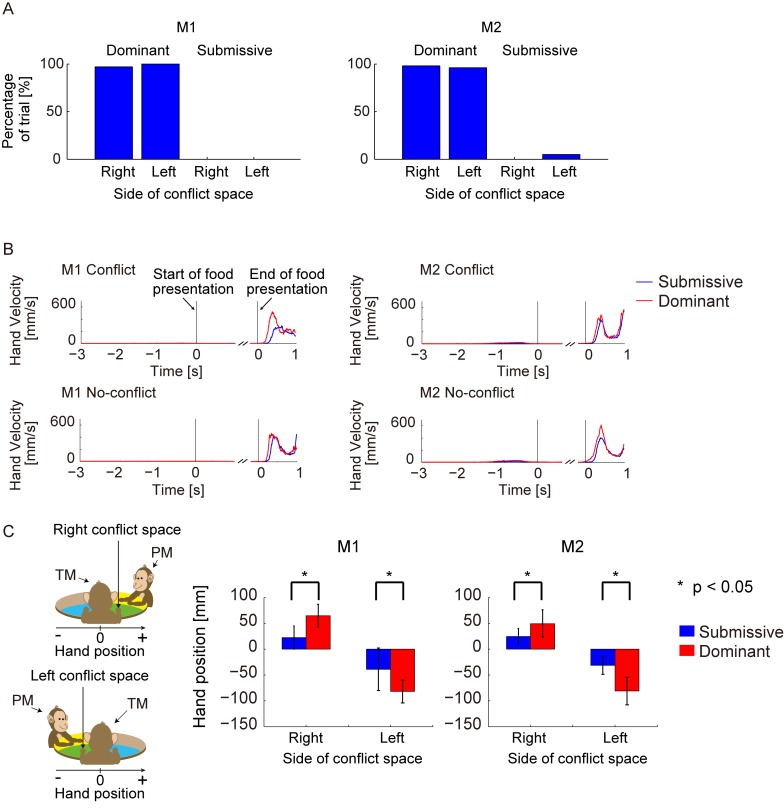
Behavioral evidence for social adaptation. A. The bars indicate the rates for each trial in which the TM tried to reach the food when the food was placed in the conflict space in the dominant and submissive conditions. When the conflict space was to the right or left side of the TM, the TM used its right or left hand, respectively, to reach for the food. The percentages of trials in which the TM moved its right or left hand toward the right or left conflict space are displayed in a bar graph for each social condition. B. Velocity of hand movement of the TM was plotted for the conflict (upper figure) and no-conflict (lower figure) conditions and for the submissive (blue line) and dominant (red line) conditions. The x-axis indicates time. Time zero on the left side of each panel shows the relative time around the start of the food-presentation period, and time zero on the right side of each figure shows the relative time to the end of the food-presentation period. Around the start and end of the food-presentation period, the velocities of the TM’s reaching motion were pooled for both the left and right arms for each conflict and no-conflict condition and for each submissive and dominant condition. Around the end of the food-presentation period in the conflict condition, we pooled only the velocities of the right and left hands used by the TM to reach food in the no-conflict space on the TM’s side. Around the end of the food-presentation period in the no-conflict condition, we pooled the velocities of the right and left hands used by the TM to reach food on the right and left sides of the TM. The median pooled values were calculated as the velocity of hand movement at that point. C. Bar graph showing the average relative hand position of the TMs on the side of the conflict space during the time window around the start of food presentation from –3000 ms to 1000 ms for M1 and from –3000 ms to 625 ms for M2. In the right- or left-side condition, the mean value of the position of the right or left hand was calculated for each social condition. The axis of the hand position was set to be parallel to a line perpendicular to the line from the center of the TM’s body to the center of the table. Error bars indicate the SDs of the hand position.

The motion-capture and video data provided three lines of evidence for the effects of hierarchical manipulations. The first line of evidence was that the submissive TM did not reach for the food in the conflict space. By using motion-capture data of the TM’s hand movement, we calculated the proportion of trials in which the TM moved its hand toward food in the conflict space when the food was placed in the right and left conflict spaces in the dominant and submissive conditions. The proportions of trials in which movement occurred in the dominant condition were 97% (M1, right), 100% (M1, left), 98% (M2, right), and 96% (M2, left). These findings suggested that the TM did not hesitate to take food in the conflict space in the dominant condition. By contrast, the proportions of trials in which movement occurred in the submissive condition were 0% (M1, right), 0% (M1, left), 0% (M2, right), and 5% (M2, left) (Fig 4A, blue bars). This indicated that the TM abandoned attempts to obtain the food in the conflict space in the submissive condition. The differences between these figures suggested that the monkey applied a completely different behavioral strategy depending on the social condition.

As the second line of evidence, the reaction times for food taking in the no-conflict space on the TM’s side were affected by the social hierarchical conditions. The reaction time was defined as the time between the end of the food-presentation period and the start of the monkey’s reaching motion when the food was placed into the no-conflict space on the TM’s side. The reaction times of the trials in which the food was placed into the no-conflict space on the TM’s side in the conflict condition and trials in which the food was placed on the TM’s side in the no-conflict condition were pooled separately, and their median values were calculated. In the conflict condition, the median reaction times were 333 ms (M1, submissive condition), 225 ms (M1, dominant condition), 258 ms (M2, submissive condition), and 209 ms (M2, dominant condition). In the no-conflict condition, the median reaction times were 300 ms (M1, submissive condition), 226 ms (M1, dominant condition), 258 ms (M2, submissive condition), and 158 ms (M2, dominant condition). In both the conflict and no-conflict conditions, the reaction time was significantly longer (Mann–Whitney *U* test, *p* < 0.05) in the submissive condition. The difference between the reaching behaviors was also confirmed by the rising timing of the TM’s hand velocity in the movement period, which was shorter in the dominant than in the submissive condition (Fig 4B).

As the third line of evidence, we observed the biased posture by looking at the TM’s hand position at the side of the conflict space. There was a significant spatial bias in the hand position between the submissive and dominant conditions (Fig 4C, *t* test, *p* < 0.05). The TM’s hand position was closer to its body center (further from the conflict space) in the submissive condition, but further from its body center (closer to the conflict space) in the dominant condition. The difference in the hand position between the submissive and dominant conditions may reflect the degree of the TM’s hesitation to take food.

**Fig 5:**
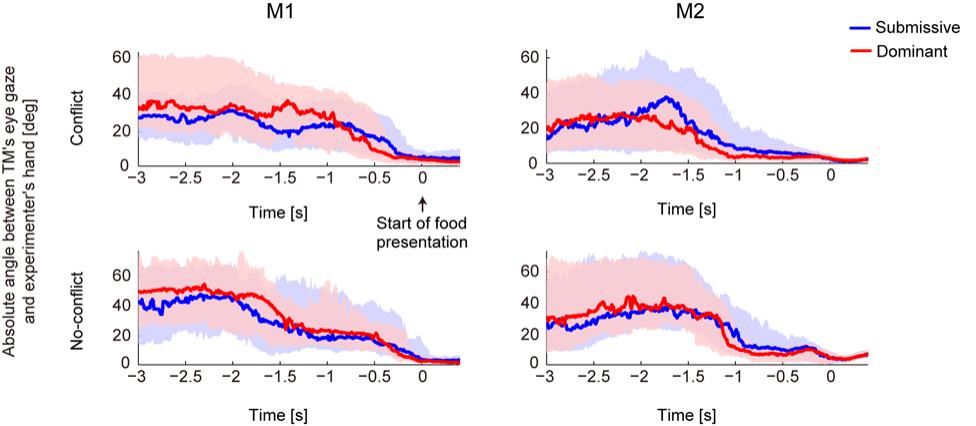
Temporal changes in eye gaze of the TM. The absolute values of the horizontal visual angle between the TM’s eye gaze and the top of the experimenter’s right hand were calculated for the conflict and no-conflict conditions for monkeys M1 and M2. In the conflict condition, the trials for the right and left conflict spaces were combined into one condition. The experimenter put his right hand under the table during the waiting and food-preparation periods, and the experimenter’s hand appeared above the table and the food was held in the right hand during the food-presentation period. The smaller angle around the start of the food-presentation period means that the TM always looked at the food. The blue and red lines represent the median angle for the submissive and dominant conditions, respectively. The lower and upper values of the shaded areas around the plots show the first and third quartiles of the angles, respectively.

These behavioral differences confirmed that the TMs changed their behavioral mode according to the PMs’ identity. In the submissive condition, the TM’s reaction time when reaching for food was slow. However, the eye-orientation data confirmed that the TMs did not ignore the food during the submissive condition in the food-presentation period (Fig 5). In all conditions including the submissive condition, the TM stared at the food regardless of social conditions.

These results suggest the existence of a social adaptive mechanism that modulates the behavioral mode. The mode appears to be maintained within each social condition, but can change if the social condition changes. We emphasize that the social hierarchy between the two monkeys was established spontaneously during the preliminary experiment. Thus, the neural representation of the social behavioral adaptation found in this task might be similar to that in natural social adaptive behaviors.

### Neural Contribution of Discriminating Social Conditions

Classifiers were generated to discriminate the social conditions (submissive vs dominant) and the reward outcome by using the full set of ECoG spectrum data during the period 3000 ms prior to 375 ms after the start of the food-presentation period. Table 1 shows numbers of trials in different social conditions for each monkey, and Table 2 shows the accuracy of the social classifier, reward expectation classifier, and chance level of these classifiers. The result shows the accuracy of the social classifier was higher than that of the chance level and the accuracy of the social classifier was much higher than that of the reward expectation classifier. These results suggest that the neural signal was more representative of social condition than reward expectation.

**Table 2:** The results of social condition classifier, reward expectation classifier and chance level of these classifiers.

Subject		Social condition	Reward expectation
**M1**	Accuracy (%)	79.58%	62.62%
**M1**	Chance level	55.01%	58.48%
**M2**	Accuracy (%)	73.98%	55.35%
**M2**	Chance level	58.16%	57.65%

**Fig 6:**
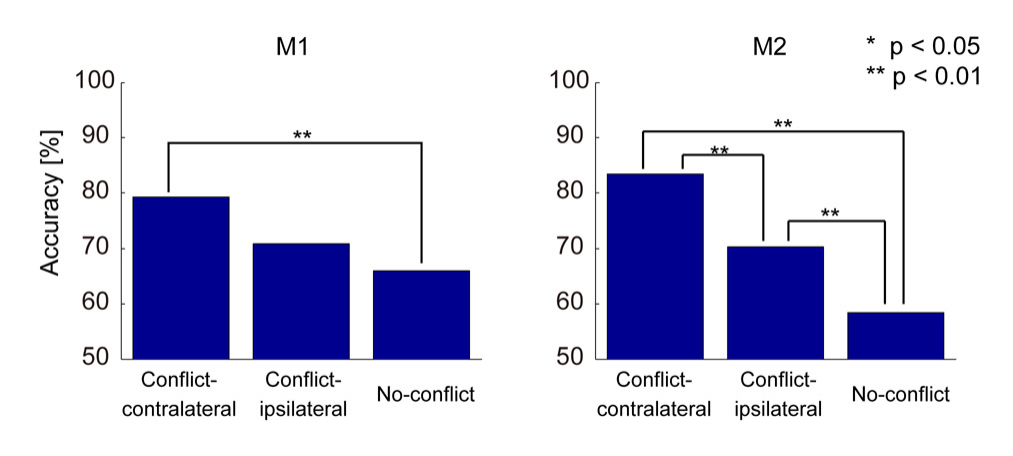
Accuracy of the full-set neural classifier. The bar graphs indicate accuracy of the full-set neural classifier in the conflict–contralateral, conflict–ipsilateral, and no-conflict conditions for monkeys M1 and M2. The classifiers were computed by using the full set of ECoG spectrum data during the period from 3000 ms prior to 375 ms after the start of the food-presentation period. We tested the differences (accuracy/chance level) between each condition to ignore the differences in chance levels for the neural classifier in each condition. In this result, there were significant differences in accuracy between the conflict–contralateral condition and the no-conflict condition in M1 and M2 (M1, Z = 2.713, p < 0.01; M2, Z = 7.115, *p* < 0.01) and between the conflict–contralateral condition and the conflict–ipsilateral condition in M2 (Z = 2.764, *p* < 0.01).

The social classifiers were developed for each conflict–contralateral, conflict–ipsilateral, and no-conflict condition. Fig 6 shows the accuracy of the classifiers by using the full set of ECoG spectrum data during the period from 3000 ms prior to 625 ms after the start of the food-presentation period for the conflict–contralateral, conflict–ipsilateral, and no-conflict conditions. A chi-square test for independent variables was conducted to show significant differences between each ratio of a condition’s neural classifier accuracy to its chance level. The chi-square test for independent variables is a well-known parametric test. The probability distribution of the accuracy must be binomial. The statistics are illustrated in Fig 6. We found a significant difference in accuracy between the conflict–contralateral condition and no-conflict conditions (in M1, Z = 2.713, *p* < 0.01, and in M2, Z = 7.115, *p* < 0.01) and the tendency of the results (conflict–contralateral condition > conflict–ipsilateral condition > no-conflict conditions) was similar in both monkeys.

This result suggested the following: (1) the ECoG could extract neural information related to the social hierarchical condition; (2) the ECoG data were more informative about the social conditions under the conflict condition than under the no-conflict condition; and (3) the ECoG data under the conflict–contralateral condition were more informative than those under the conflict–ipsilateral condition. Because the conflict–contralateral condition showed the highest accuracy, we focus on this condition in the following analysis.

**Fig 7:**
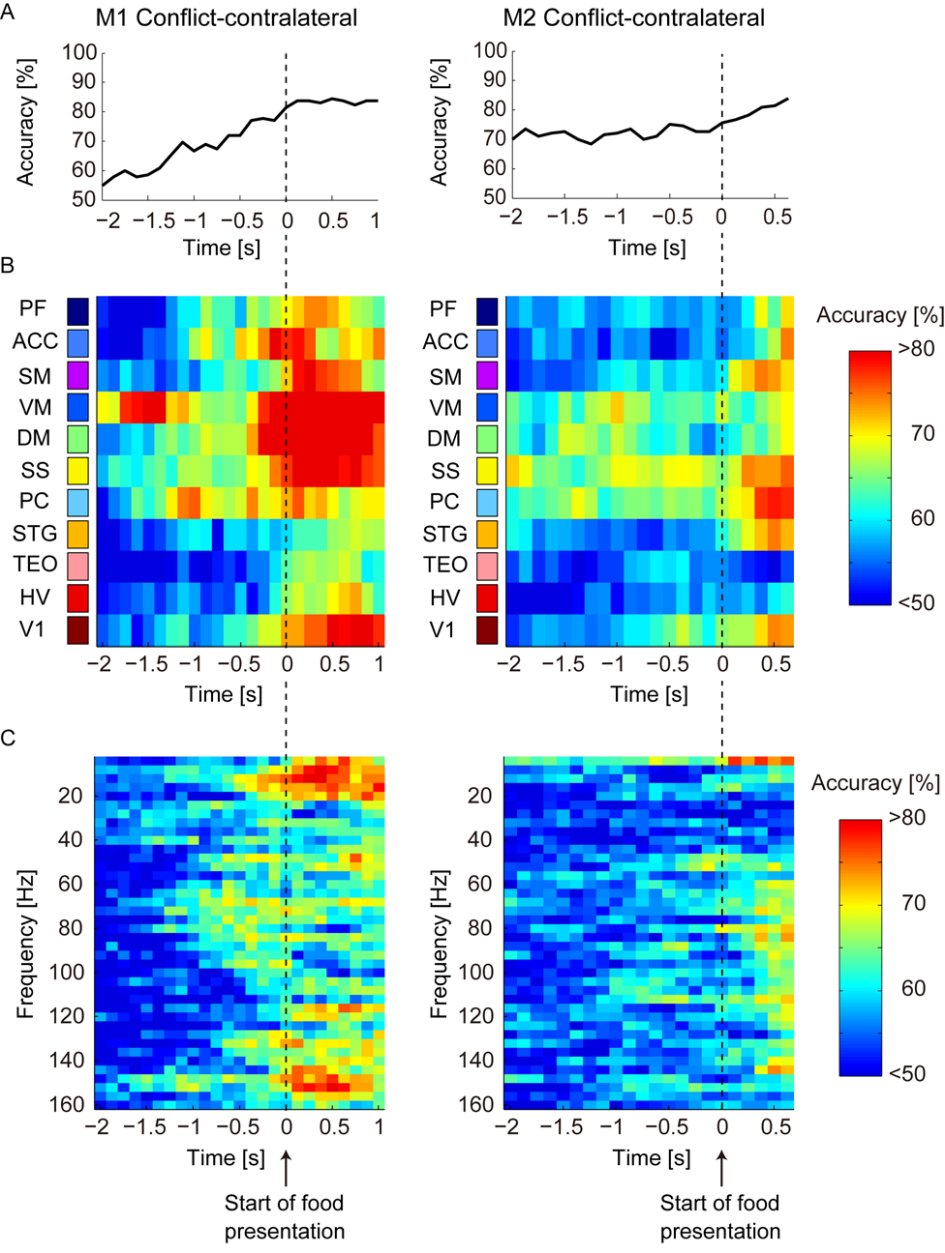
Accuracies of the temporal, spatiotemporal, and spectrotemporal neural classifiers. All figures are aligned to the start of the food-presentation period (vertical dashed lines). The range of time intervals used in the analysis was –3000 ms to 1000 ms for M1 and –3000 ms to 625 ms for M2. The numbers on the x-axis indicate the end of each long time bin. A: Temporal sequence of the accuracy of the temporal neural classifier in the conflict–contralateral condition. B: Temporal sequence of the accuracy of the spatiotemporal neural classifier in the conflict–contralateral condition. The color of the bars represents the accuracy of the classifier. C: Temporal sequence of the accuracy of the spectrotemporal neural classifier in the conflict–contralateral condition. The color of the bars represents the accuracy of the classifier.

We searched for the brain region that determined which temporal timing and frequency band discriminated the social conditions. We decomposed the full set of ECoG sensor data into three subsets of ECoG features (Fig 3). We constructed three types of neural classifier: temporal, spatiotemporal, and spectrotemporal. The results of these neural classifiers explain how the information about social conditions was distributed in the feature space of the ECoG data. Fig 7A shows that the accuracy of the temporal classifier gradually increased over time during the food-preparation and food-presentation periods. During these periods, the accuracies reached 80% in both M1 and M2. The spatiotemporal neural classifier showed a specific spatiotemporal pattern (Fig 7B) of accuracy that could discriminate the social conditions. The cortical region that showed the greatest accuracy was localized in the parietal and somatosensory–motor cortices (PC, SS, VM, and DM) before the appearance of food, and this region expanded to the visual and prefrontal cortices (V1, PF, ACC, and SM) after the appearance of food. These trends were similar in the two monkeys.

The spectrotemporal neural classifier showed (Fig 7C) that the accuracy of the neural classifier increased sparsely across frequency ranges during the food-preparation period and tended to increase across wide frequency ranges after the onset of the food-presentation period (Fig 7C). There was no obvious common distribution pattern in the frequency domain in M1 and M2.

These findings showed that there was consistency across individuals in the temporal and spatiotemporal neural classifiers, but that there was less individual consistency in the spectrotemporal neural classifier. This suggests that the information about social hierarchy is localized spatiotemporally. In the following analysis, we focus on the detailed spatiotemporal pattern of accuracy obtained from the spatiotemporal neural classifier.

### The Sequential Appearance of Information on Social Hierarchy on Cortical Regions

**Fig 8:**
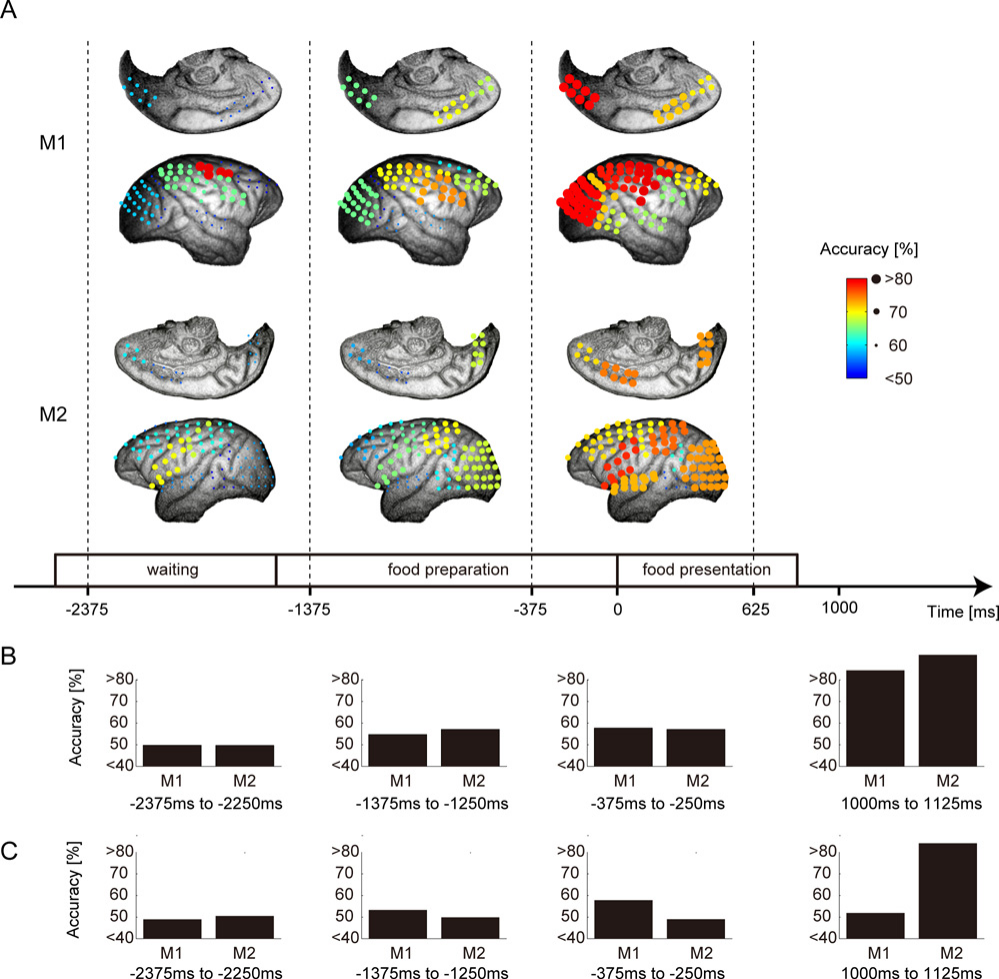
Comprehensive spatiotemporal pattern of the representation of social conditions. A: The spatial distribution of the accuracy of the spatiotemporal neural classifier in the conflict–contralateral condition for monkeys M1 and M2. The size and color of each circle are proportional to its accuracy. Three successive long time bins were selected from the waiting, food-preparation, and food-presentation periods (left, middle, and right). Dashed lines indicate the boundary of the long time bins. Numbers are shown relative to the start time of the food-presentation period under the time line indicating the start and end time of each long bin. B: The accuracy of the temporal neural classifier that discriminated whether the monkey used its right or left hand during the movement period for monkeys M1 and M2 during four time windows. C: The accuracy of the classifier that discriminated reward outcome from neural activity and outcomes of the preceding three trials during four time windows.

The accuracy of the spatiotemporal neural classifier was mapped on the brain image to illustrate more comprehensively the spatiotemporal pattern of the representation of social conditions (Fig 8A). During the waiting and early food-preparation periods, when the food did not appear and the competition was still implicit, the TM’s hand position differed significantly between social conditions (Fig 4C), indicating that the effect of social status had started and was maintained across trials. The accuracy was already high and the representation was localized mainly in the parietal and somatosensory–motor cortices (Fig 8A, left column). During the following food-preparation period when the experimenter prepared the food under the table, the visual, high-order motor, and prefrontal cortices gradually started to discriminate the social conditions (Fig 8A, middle) and the accuracy was sustained. During the food-presentation period when the food appeared and the competition became explicit, the accuracy remained higher in most of the cortical regions (Fig 8A, right). These results indicate that the information about discriminating social hierarchical conditions spread gradually and progressively over the entire cortical surface as the social conflict became more explicit. Note that the monkey could not prepare an action plan during these epochs because there was no available information about a location of reaching goal. To dissociate the motor preparation from the social condition, we applied two decoder analyses. Fig 8B shows the accuracy of the motor action decoder in three epochs. The hand movement classifier was constructed to predict which arm the monkey would use during the reaching period. The decoder showed high predictive performance in the movement period, but not before the food-presentation period. This result suggested that the monkey did not prepare a detailed motor plan during these periods.

Although, the monkey did not appear to prepare a precise motor plan before food presentation, it was still possible to show social context-dependent modulation of attention, arousal, and motivation. These parameters are highly subjective, so it is not easy to quantify and control them under a natural social setting. Instead of directly measuring these parameters, we referred to the outcomes of the previous three trials as a reflection of these parameters. If a monkey obtains a food reward in each of the previous three trials, its food expectation will be high, therefore attention, arousal, and motivation levels must be high. Conversely, when a monkey fails to obtain a food reward for three successive trials, its expectation will be low, therefore attention, arousal, and motivation levels must be low. Fig 8C shows the results of the food expectation decoder during four epochs before and after food presentation that predicts outcomes of the trial from neural activity and the history of the previous three trials. The prediction performance was below 60% before food presentation, which suggested that reward expectation was not much represented during the periods.

## Discussion

In this study, we found that the relationship between the specific spatiotemporal neural pattern and social suppressive behavior was more obvious in the contralateral condition, and the neural pattern was not contaminated much by reward expectation and related subjective internal states. Abstract information about social behavioral control in the frontal cortex observed in this study might reflect the higher-order cognitive process that suppresses an impulsive response to the food under the submissive condition. The similar neural modulation can be observed during preparatory period as a simple form of motor preparation/execution processes under non-social setting. Our finding suggests that there is higher-order cognitive mechanism that can regulate the motor control mechanism studied previously under non-social setting.

Conventionally it is thought that motor control is achieved by multiple steps; 1) motivation/intention > 2) action planning/evaluation > 3) decision making > 4) motor preparation > 5) motor execution > 6) outcome evaluation > 7) action completion [21]. These functional steps tended to be assigned to multiple cortical areas and sequential activation starts from rostral part of frontal lobe and expand to caudal part. Neural mechanism of rostro-caudal sequential processing has been studied mostly in non-social setup. In contrast, there is no stable delay/preparatory period (step 2–4 in above that are often set in conventional behavioral task) in natural social setting, because social context is fragile and instantly influenced by others’ behavior that requires momentary behavioral response. In our food grab task, social context is relatively stable so that during waiting, food preparation, food show and food delivery periods, monkey could start action preparation in accordance with current social context. This tells that motor preparation/execution mechanism could be potentially involved in the neural modulations at any period during the performance of the social food-grab task as an underlying neural basis.

Generally, information about cognitive function is presumed to be localized in a specific frequency band [22]. By contrast, our findings suggest that the combination of the power spectrum in the broadband frequency enabled the neural classifier to extract a stable and unique representation of social information in the spatial and temporal dimensions (Fig 7A and Fig 7B), even though there was an individual difference in the frequency domain (Fig 7C). One possible interpretation of the individual difference is that the difference in the frequency domain reflected a difference in how cortical regions communicate with each other, even if the participating regions are the same [23]. In the following discussion, we focus on the common spatiotemporal patterns between subjects in the prefrontal, V1, and parietal and somatosensory–motor areas during the delay period before motor preparation had started.

ECoG data in the prefrontal area could discriminate the social hierarchical condition during the food-presentation period not during preceding the waiting and food-preparation period. Since prefrontal cortex guides action sequence as executive system and there was little performance of discriminating social hierarchical conditions in prefrontal cortex, motor preparation/execution sequence was not yet much started during the waiting and food-preparation periods (see Fig 8B). Fujii et al. showed that the baseline activity level of neurons in the prefrontal cortex remained high in the dominant condition and was suppressed in the submissive condition [3]. They noted that the prefrontal cortex is a candidate area that may generate and retain the neural representation of the social conditions when the social behavioral mode is sustained throughout the task. However, our findings suggest that the prefrontal regions had less ability to retain information about social conditions during the period when the food was invisible (Figs 7B and 8). The difference between the previous study [3] and this study might relate to the duration of the trials. The mean duration of one trial during the food-grasp task was 5 s in the previous study and 14 s for M1 and 11 s for M2 in our study. The main difference between the previous study and our study is that in our study the experimenter waited until the monkey had finished eating the food. In our study, the average intervals between reward timing (when the monkey started to eat the food) in a previous trial and the onset of the food-presentation period in the next trial were 11 s for M1 and 9 s for M2. In the previous study, neural activity in the prefrontal cortex showed sustained representation of the social condition because of the continuous cortical state, but in our study this was observed mainly after the appearance of food. It has been hypothesized that the prefrontal cortex is involved more in the change in context [24,25] than in maintaining the context. Our finding seems to support this hypothesis because the prefrontal cortex represented the social contextual information during the period when social competition became explicit and when the monkey was required to make a behavioral decision [26,27].

During the period before food presentation (early context period), the information about social context was created to enable execution of the cognitive decision-making process that followed. In the natural social environment, this social decision function is acquired as a control of impulsivity through development. Shannon et al. found that the high correlation between blood oxygen level-dependent activity in the premotor region and that in the brain networks associated with spatial attention and executive control (frontal eye fields, dorsolateral prefrontal cortex, and anterior cingulate cortex) regulates impulsivity in juveniles [11]. This suggests that the maturation of the connection of the attention and executive control network with the premotor cortex is involved in the self-regulation of impulsivity. It also suggests that it is not possible to dissociate pure social adaptation mechanism from motor control (preparation and execution processes) because of the developmental origin. Social adaptive mechanism is acquired through development as a self-regulation system that can override impulsive motor response.

The contributions of the premotor and parietal cortices during the early context period in the social conditional classification may suggest a possible contribution of the mirror neuron system [28,29]. However, during these periods, the monkeys showed almost no goal-directed actions, and this is unlikely to explain the contribution of the mirror neuron system in the social conditional classification.

The V1 area started to discriminate the social condition after the onset of the food-presentation period (Figs 7B and 8). Because the TM stared at the food around the onset of the food-presentation period independent of the submissive or dominant condition (Fig 5), the difference in social contextual representation in the V1 area may reflect the input of cognitive processes, such as attention to the food [30]. Even though the TM saw the food in both conditions, it is possible that it observed the food more carefully in the dominant condition because the probability of acquiring the food was higher in the dominant condition than in the submissive condition, and the higher expectation for the food may induce greater recruitment of the attention mechanism as a top-down modulation [31,32].

The parietal and somatosensory–motor cortices could discriminate the social hierarchical conditions before and after the onset of the food-presentation period (Figs 7B and 8A). We note that we did not analyze the food-reaching period to dissociate the influence of motor execution. The context periods we analyzed were the waiting, food-preparation, and food-presentation periods in which the monkey waited for the food delivery, and these had minor effects on motor control (Fig 4B). By contrast, the significant difference in the TM’s hand position between the submissive and dominant conditions showed that social context was maintained during these periods (Fig 4C).

A straightforward interpretation of why these cortical regions could discriminate social hierarchy lies in the difference in motor preparation/intention under different social conditions. It seems natural to think that the TM was less motivated in the submissive condition, but was eager in the dominant condition. The difference in the monkey’s intention might lead to a difference in motor preparation/intention, even though there was no observable difference in the motor behavior between the dominant and submissive conditions (Fig 4B) and no future motor planning was represented in the regions (Fig 8B). Reward expectation was also tested using recent reward acquisition history, but the activity pattern did not reflect the preparation/intention level during these periods (Fig 8C). The smaller contribution of the prefrontal cortex during the early context period indicates that, once the rank of social hierarchy was established between the monkeys, the prefrontal cortex was not required to sustain the social contextual information, but the information could be sustained as an embodiment representation observed in the monkey’s posture, which is controlled by the parietal and somatosensory–motor cortices. This suggests that social adaptation is achieved by a higher-order decision process in accordance with the embodied social contexts during earlier context period [13] followed by releasing motor preparation and execution processes during later context period and movement period.

We suggest that the change in spatiotemporal pattern representing the information about the social condition in multiple cortical regions reflects the adaptive behavioral-modulation process that produces social suppressive behavior. In this study, we have shown the social adaptive neural mechanism at the activity level, but it remains unclear how this function is achieved at the dynamic network level [32]. Further understanding of the mechanisms underlying the functional network of social adaptive behavior will require new analytical methods to visualize the dynamic information processing of the large-scale network.

## Supporting Information

S1 Text The ARRIVE Guidelines Checklist. (DOC)
