# Supplementary material for: Social Suppressive Behavior Is Organized by the Spatiotemporal Integration of Multiple Cortical Regions in the Japanese Macaque
Source: PLoS One. 2016 Mar 10;11(3):e0150934. doi: 10.1371/journal.pone.0150934 (PMC4786196; doi:10.1371/journal.pone.0150934)
Supplement: S1 Text — (DOC) [file pone.0150934.s001.doc]

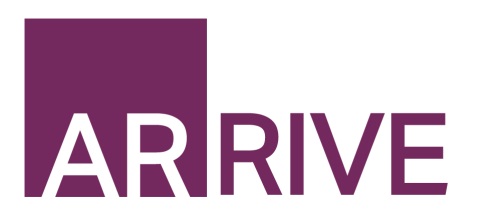


The ARRIVE Guidelines Checklist

Animal Research: Reporting In Vivo Experiments

Carol Kilkenny^1^, William J Browne^2^, Innes C Cuthill^3^, Michael Emerson^4^ and Douglas G Altman^5^

*^1^The National Centre for the Replacement, Refinement and Reduction of Animals in Research, London, UK, ^2^School of Veterinary Science, University of Bristol, Bristol, UK, ^3^School of Biological Sciences, University of Bristol, Bristol, UK, ^4^National Heart and Lung Institute, Imperial College London, UK, ^5^Centre for Statistics in Medicine, University of Oxford, Oxford, UK.*

|  | ITEM | RECOMMENDATION | Section/ Paragraph |
| --- | --- | --- | --- |
| Title | 1 | Provide as accurate and concise a description of the content of the article as possible. | Social suppressive behavior is organized by the spatiotemporal integration of multiple cortical regions in the Japanese macaque |
| Abstract | 2 | Provide an accurate summary of the background, research objectives, including details of the species or strain of animal used, key methods, principal findings and conclusions of the study. | BACKGROUND AND PURPOSE:  Under social conflict, monkeys develop hierarchical positions through social interactions. Once hierarchy is established, the dominant monkey dominates the space around itself and the submissive monkey tries not to violate this space. Previous studies have shown the contributions of the frontal and parietal cortices in social suppression, but the contributions of other cortical areas to suppressive functions remain elusive.  EXPERIMENTAL APPROACH:  We recorded neural activity in large cortical areas using electrocorticography (ECoG) arrays while monkeys performed a social food-grab task in which a target monkey was paired with either a dominant or submissive monkey. If the paired monkey was dominant, the target monkey avoided taking food in the shared conflict space but not in other areas. By contrast, when the paired monkey was submissive, the target monkey took the food freely without hesitation.  KEY RESULTS:  We applied decoding analysis to the ECoG data and clarified how much information about the social condition was represented. Information about the social condition was modulated according to the conflict space location. More information about the social conditions was found when the conflict space was set in the hemisphere contralateral to the recording hemisphere. We also found that the information increased as the social pressure increased during the task. Before food presentation when the pressure was relatively low, the parietal and somatosensory–motor cortices showed sustained discrimination of the social condition. After food presentation when the monkey was faced with greater pressure to make a decision whether he should take the food, the prefrontal and visual cortices started to develop build-up responses. The social representation was found in a sustained form in the parietal and somatosensory motor regions, followed by the build-up form in the visual and prefrontal cortices.  CONCLUSIONS AND IMPLICATIONS:  These findings suggest that the social adaptive mechanism is achieved by temporally different integration of multiple cortical regions. |
| INTRODUCTION | | |  |
| Background | 3 | a. Include sufficient scientific background (including relevant references to previous work) to understand the motivation and context for the study, and explain the experimental approach and rationale.  b. Explain how and why the animal species and model being used can address the scientific objectives and, where appropriate, the study’s relevance to human biology. | a-b.  Human beings are social animals that manipulate behavioral repertoires depending on the social contexts generated by integration of environmental conditions and an individual’s past social experiences. We acquire this social adaptive intelligence throughout the developmental period. Social adaptation is a subjective internal process that is unique for each individual, and this process may differ between individuals. However, regardless of the uniqueness of the internal mechanism, the expression form of our social behavior is similar; therefore, there must be a common adaptive mechanism within the brain.  In monkey society, social adaptive behavior is also essential and can be observed as suppressive behavior. When two monkeys are paired and share the same space, one monkey suppresses its action repertoires within the space to avoid violating the other monkey’s space. This suppressive function for avoiding social conflict is important to maintaining a stable social society [1]. To achieve this social suppressive behavior, a monkey must consider various social factors such as its own intentions and those of others [2], past experiences with others, its hierarchical status [3,4], what others are paying attention to, and the relative distance between individuals [5]. The monkey must integrate these social factors, called the social context. Failure to integrate these social factors will lead to inappropriate social behavior.  Autistic humans often fail to perceive social information, such as emotion and intention, from others’ faces, which leads to disordered social communication [6,7]. To ensure appropriate social behavior, we must control our impulsivity during social interactions. It has been suggested that the prefrontal cortex is involved in the inhibitory control of impulsivity [8-10]. At the network level, a study of impulsivity in juveniles has shown that the strength of the connection between the premotor cortex and attention network including the prefrontal cortex correlated with the controllability of impulsive behavior [11]. This finding suggests that we should consider the neural activity in the global brain network when trying to understand the neural mechanisms responsible for social suppressive behavior.  In previous studies of social suppressive behavior in the monkey, we recorded neural activity from the parietal [4] and prefrontal [3,4] cortices and caudate nucleus [12] while monkeys performed a social food-grab task (see Figure 1A). In these studies, the monkeys were not restrained much so that they could interact freely and show social adaptive behavior. Neurons recorded from different areas exhibited different neural properties during the task. In the caudate nucleus, we found social context-dependent neural activity during the food-show period before the food-taking period of the social food-grab task [12]. Some neurons in the caudate nucleus exhibited increased neural activity during the food-show period, but the increased activity was suppressed when the monkey showed submissive behavioral suppression. By contrast, the prefrontal baseline activity exhibited sustained context-dependent modulation [3]. These prefrontal neurons showed tonic baseline activity in the socially free condition, which did not require social adaptation, but showed a significant decrease or increase in baseline activity in the submissive or dominant mode, respectively. These findings suggest that adaptive social behavior is achieved by social context-dependent neural modulation. |
| Objectives | 4 | Clearly describe the primary and any secondary objectives of the study, or specific hypotheses being tested. | These previous studies revealed that selected cortical and subcortical regions are involved in social context-dependent adaptive behavior. However, we do not know how the other areas are involved in this process or how social context is generated, represented, and used. To perform the food-grab task in a socially appropriate manner, monkeys must consider who the paired monkey (PM) is, its past experience with the PM, the relative location of the PM, and the PM’s intention at that moment. We hypothesized that this information is integrated and represented as social context in the brain and is used as a reference for adaptive social behavior [13]. To investigate this idea further requires recording and analyzing the neural activity from large cortical areas simultaneously. By using electrocorticography (ECoG) arrays, we recorded neural activity from most of the lateral cortical surface and a part of the medial prefrontal cortex while monkeys performed a social food-grab task. We found that the spatiotemporal patterns of neural dynamics correlated with social suppressive behavior. |
| METHODS | | |  |
| Ethical statement | 5 | Indicate the nature of the ethical review permissions, relevant licences (e.g. Animal [Scientific Procedures] Act 1986), and national or institutional guidelines for the care and use of animals, that cover the research. | The experimental protocols in the experiment and surgery were approved by the RIKEN ethics committee (No. H24-2-203(4)) and were in accordance with the recommendations of the Weatherall report “The use of non-human primates in research.” Implantation surgery was performed under sodium pentobarbital anesthesia, and all efforts were made to minimize suffering. No animal was sacrificed in this study. Overall animal care was managed by the Division of the Research Resources Center at RIKEN Brain Science Institute. |
| Study design | 6 | For each experiment, give brief details of the study design including:  a. The number of experimental and control groups.  b. Any steps taken to minimise the effects of subjective bias when allocating animals to treatment (e.g. randomisation procedure) and when assessing results (e.g. if done, describe who was blinded and when).  c. The experimental unit (e.g. a single animal, group or cage of animals).  d. A time-line diagram or flow chart can be useful to illustrate how complex study designs were carried out. | In the food-grab task, we manipulated two conditions: 1) the social hierarchy between the TM and PM and 2) the relative locations of the two monkeys. We prepared two TMs (M1 and M2, Macaca fuscata). Age and weight of M1 and M2 were 9 years old and 8.8 kg, and 6 years old and 8.8 kg respectively. To manipulate the TM’s social hierarchical (submissive or dominant) status, each TM had two PMs. We prepared three monkeys who were not implanted with ECoG arrays as the PM (M3–M5, Macaca fuscata). Age and weight of M3-M5 were 8 years old and 11.7 kg, 4 years old and 7.1 kg and 10 years old and 10.8 kg respectively. For M1, M3 was paired as the dominant monkey and M4 was paired as the submissive monkey. For M2, M5 was paired as the dominant monkey and M4 was paired as the submissive monkey. The hierarchical ranks were: M3 > M1 > M4 and M5 > M2 > M4.  b.  Cross-validation test was applied to all results of decoder:  We used a support vector machine (L2 norm, linear kernel, C value set to 1) as the neural decoder and evaluated its performance using a 50-fold cross-validation test [17].  c.  The food-grab task involved one human experimenter and two monkeys: a target monkey (TM), whose neural activity was recorded, and a PM.  d.  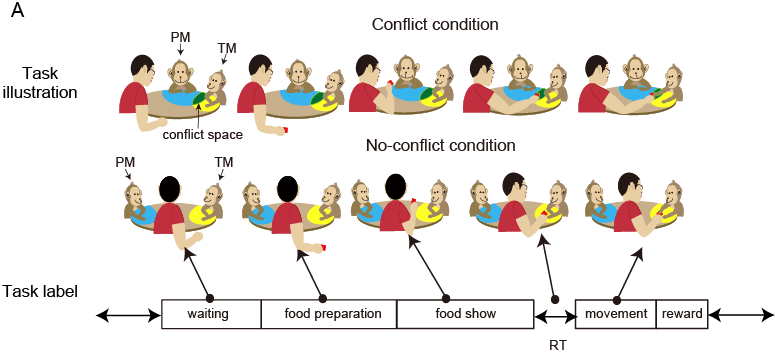  Figure 1 A:Task illustration: Schematic sequence of the food-grab task in the conflict and no-conflict conditions. The task had five periods: waiting, food preparation, food show, movement, and reward. TM, target monkey; PM, paired monkey. The spaces around the monkeys that were reachable only by the TM and PM are shown in yellow and blue, respectively. Green represents the conflict space, which was shared by the TM and PM. Task label: The labels of five periods are shown under the Task illustration. RT, reaction time between the end of the food-show period and the onset of the movement period. |
| Experimental procedures | 7 | For each experiment and each experimental group, including controls, provide precise details of all procedures carried out. For example:  a. How (e.g. drug formulation and dose, site and route of administration, anaesthesia and analgesia used [including monitoring], surgical procedure, method of euthanasia). Provide details of any specialist equipment used, including supplier(s).  b. When (e.g. time of day).  c. Where (e.g. home cage, laboratory, water maze).  d. Why (e.g. rationale for choice of specific anaesthetic, route of administration, drug dose used). | a.  Implantation surgery was performed under sodium pentobarbital anesthesia, and all efforts were made to minimize suffering.  b-c.  All experiments were done in an experimental laboratory during daylight.  d.  None |
| Experimental animals | 8 | a. Provide details of the animals used, including species, strain, sex, developmental stage (e.g. mean or median age plus age range) and weight (e.g. mean or median weight plus weight range).  b. Provide further relevant information such as the source of animals, international strain nomenclature, genetic modification status (e.g. knock-out or transgenic), genotype, health/immune status, drug or test naïve, previous procedures, etc. | a.  It is the same as 6-a.  b.  In this study, we used five monkeys. The two monkeys were provided by NBRP “Japanese Monkeys” through the National BioResource Project of the Ministry of Education, Culture, Sports, Science and Technology (MEXT), Japan. Other three monkeys were provided by Hamri co., Ltd. (Ibaragi, Japan). |

The ARRIVE guidelines. Originally published in *PLoS Biology*, June 2010^1^

| Housing and husbandry | 9 | Provide details of:  a. Housing (type of facility e.g. specific pathogen free [SPF]; type of cage or housing; bedding material; number of cage companions; tank shape and material etc. for fish).  b. Husbandry conditions (e.g. breeding programme, light/dark cycle, temperature, quality of water etc for fish, type of food, access to food and water, environmental enrichment).  c. Welfare-related assessments and interventions that were carried out prior to, during, or after the experiment. | | a-c.  Each animal was housed in an individual cage with other animals visible and was maintained on a 12:12-h light:dark cycle with lights on at 8:00am in a temperature (25±2°C) and humidity (50±20%) controlled room. The dimensions of the cage size were H 130cm × W 70cm × D 85cm. The animals were given food (PS-A; Oriental Yeast Co., Ltd., Tokyo, Japan) and water ad libitum, and daily fruit/dry treats as a means of enrichment and novelty. After every experiment, gummy candies and nuts were given as treats. The animal was occasionally provided toys in the cage. The in-house veterinary doctor checked the animals and updated daily feedings to maintain weight. We attempted to offer as humane treatment of our subjects as possible. | |
| --- | --- | --- | --- | --- | --- |
| Sample size | 10 | a. Specify the total number of animals used in each experiment, and the number of animals in each experimental group.  b. Explain how the number of animals was arrived at. Provide details of any sample size calculation used.  c. Indicate the number of independent replications of each experiment, if relevant. | | a-b.  It is the same as 6-a.  c.  None | |
| Allocating animals to experimental groups | 11 | a. Give full details of how animals were allocated to experimental groups, including randomisation or matching if done.  b. Describe the order in which the animals in the different experimental groups were treated and assessed. | | a.  It is the same as 6-a.  We performed the food-grab experiments for several days to obtain a sufficient number of trials for the analysis. The ECoG data had good stability over days [15]. In M1’s case, the numbers of trials on each day were 242 and 248 (2 days). In M2’s case, the numbers of trials were 218, 219, and 215 (3 days). These days were successive. The trials on each day contained all six conditions: submissive or dominant and ipsilateral conflict, contralateral conflict, or no-conflict. The sequence of task conditions was randomized everyday. For each submissive and dominant condition, the sequential order of ipsilateral conflict, contralateral conflict and no-conflict conditions were randomized. All experiments were performed in the experimental laboratory during daytime. | |
| Experimental outcomes | 12 | Clearly define the primary and secondary experimental outcomes assessed (e.g. cell death, molecular markers, behavioural changes). | | Behaviour data:  The monkeys’ behavior was monitored using a motion-capture system (Vicon, Oxford, UK) and a custom-made head-free eye-tracking system [15].  Neural data:  We recorded ECoG signals from two Macaca fuscata monkeys (M1 and M2). We implanted 128-channel ECoG arrays (Unique Medical, Tokyo, Japan) in the left or right hemisphere of M1 or M2. | |
| Statistical methods | 13 | a. Provide details of the statistical methods used for each analysis.  b. Specify the unit of analysis for each dataset (e.g. single animal, group of animals, single neuron).  c. Describe any methods used to assess whether the data met the assumptions of the statistical approach. | | a-c.  Reaction time:  In both the conflict and no-conflict conditions, the reaction time was significantly longer (Mann–Whitney test, p < 0.05) in the submissive condition.  Monkey’s hand position(Figure 4C):  There was a significant spatial bias in the hand position between the submissive and dominant conditions (Figure 4C, t test, p < 0.05).  Accuracy of full-set classifier (Figure 6):  The chi-square test of independence showed significant differences in accuracies between the conflict–contralateral, conflict–ipsilateral, and no-conflict conditions (p < 0.05). | |
| RESULTS | | | |  | |
| Baseline data | 14 | For each experimental group, report relevant characteristics and health status of animals (e.g. weight, microbiological status, and drug or test naïve) prior to treatment or testing. (This information can often be tabulated). | | It is the same as 6-a. | |
| Numbers analysed | 15 | 1. Report the number of animals in each group included in each analysis. Report absolute numbers (e.g. 10/20, not 50%^2^).   b. If any animals or data were not included in the analysis, explain why. | | a.  Two monkeys (M1 and M2)  b.  None | |
| Outcomes and estimation | 16 | Report the results for each analysis carried out, with a measure of precision (e.g. standard error or confidence interval). | | Monkey’s hand position (Figure 4C):  Error bars indicate the SDs of the hand position.  Temporal changes in eye gaze of the TM (Figure 5):  The lower and upper values of the shaded areas around the plots show the first and third quartiles of the angles, respectively. | |
| Adverse events | 17 | a. Give details of all important adverse events in each experimental group.  b. Describe any modifications to the experimental protocols made to reduce adverse events. | | a.  None  b.  None | |
| DISCUSSION | | | |  | |
| Interpretation/ scientific implications | 18 | | a. Interpret the results, taking into account the study objectives and hypotheses, current theory and other relevant studies in the literature.  b. Comment on the study limitations including any potential sources of bias, any limitations of the animal model, and the imprecision associated with the results^2^.  c. Describe any implications of your experimental methods or findings for the replacement, refinement or reduction (the 3Rs) of the use of animals in research. | a.  In conclusion, we recorded neural activity from most of the lateral cortical surface and part of the medial prefrontal cortex while monkeys performed a social food-grab task. We found that the amount of information related to discriminating the social context increased as the pressure of social conflict increased when the conflict space was contralateral to the recording hemisphere. Sustained social information in the parietal and somatosensory–motor cortices appeared first before the competition was implicit and was propagated to the V1 and prefrontal cortices when the competition became explicit. We suggest that the change in spatiotemporal pattern representing the information about the social condition in multiple cortical regions reflects the adaptive behavioral-modulation process that produces social suppressive behavior.  b.  None  c.  None | |
| Generalisability/translation | 19 | | Comment on whether, and how, the findings of this study are likely to translate to other species or systems, including any relevance to human biology. | None | |
| Funding | 20 | List all funding sources (including grant number) and the role of the funder(s) in the study. | | This work was supported by JSPS KAKENHI Grant Numbers 23118003 and 23650225, and “Brain Machine Interface Development” performed under the Strategic Research Program for Brain Sciences by MEXT, Japan. |  |


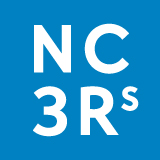


References:

1. Kilkenny C, Browne WJ, Cuthill IC, Emerson M, Altman DG (2010) Improving Bioscience Research Reporting: The ARRIVE Guidelines for Reporting Animal Research. *PLoS Biol* 8(6): e1000412. doi:10.1371/journal.pbio.1000412

2. Schulz KF, Altman DG, Moher D, the CONSORT Group (2010) CONSORT 2010 Statement: updated guidelines for reporting parallel group randomised trials. *BMJ* 340:c332.
